# Control when confidence is costly

**Published:** 2024-10-29

**Authors:** Itzel Olivos Castillo, Paul Schrater, Xaq Pitkow

**Affiliations:** Department of Computer Science, Rice University, Houston, TX 77005; Departments of Computer Science and Psychology, University of Minnesota, Minneapolis, MN 55455; Departments of Electrical and Computer Engineering and Computer Science, Rice University, Houston, TX 77005; Neuroscience Institute and Department of Machine Learning, Carnegie Mellon University, Pittsburgh, PA 15213; Department of Neuroscience, Baylor College of Medicine, Houston, TX 77030

## Abstract

We develop a version of stochastic control that accounts for computational costs of inference. Past studies identified efficient coding without control, or efficient control that neglects the cost of synthesizing information. Here we combine these concepts into a framework where agents rationally approximate inference for efficient control. Specifically, we study Linear Quadratic Gaussian (LQG) control with an added internal cost on the relative precision of the posterior probability over the world state. This creates a trade-off: an agent can obtain more utility overall by sacrificing some task performance, if doing so saves enough bits during inference. We discover that the rational strategy that solves the joint inference and control problem goes through phase transitions depending on the task demands, switching from a costly but optimal inference to a family of suboptimal inferences related by rotation transformations, each misestimate the stability of the world. In all cases, the agent moves more to think less. This work provides a foundation for a new type of rational computations that could be used by both brains and machines for efficient but computationally constrained control.

## 1 Introduction

Enabling robots to exhibit human-level performance when solving tasks in unexpected operating conditions remains a major milestone waiting to be conquered by Artificial Intelligence. One of the main reasons this milestone has yet to be achieved is the ubiquity of uncertainty in real-world applications. In realistic settings, robots interact with a continuously changing world using sensors with limited precision and actuators that become unreliable over time. Under these conditions, planning without carefully considering the long-term effects of errors in perception and mobility can be devastating. Agents need self-adaptive capabilities that mitigate uncertainty with tractable computation.

A well-known, powerful framework to solve control problems in uncertain environments is the Partially Observable Markov Decision Process (POMDP). In this framework, the agent builds and updates a belief distribution over hidden states to make informed decisions that maximize expected utility [1]. This probabilistic reasoning approach offers compelling benefits for control; e.g. it allows the agent to make decisions robust to epistemic and aleatoric uncertainty, provides the machinery to incorporate prior knowledge into the decision-making algorithm, and, compared to recurrent neural networks, is easier to interpret [2, 3]. In addition, by incorporating errors and uncertainty about the agent’s internal states into the agent’s belief, POMDPs also provide an agent the capability for self-adaptive and approximate internal control[4]. Unfortunately, POMDP’s benefits come at the cost of the high computational complexity of belief updating—a core problem that has limited its practicality in robotics problems. Here we tackle this problem by providing the agent the ability to rationally approximate inference for efficient control.

Updating beliefs in settings involving continuous state spaces, non-linear dynamics, and high-dimensional sensory data is a resource-intensive inference process. The number of operations (flops) needed to carry it out for linear, Gaussian problems is already cubic in the number of dimensions [5], and can be much harder in general [6]. Conventional approaches, such as Variational Bayes or Markov Chain Monte Carlo, propose various approximations to make inference more feasible by trading off computational costs with errors in belief. Although these approaches are widely used in control, inference approximations are generally chosen *a priori* to optimize generic belief quality measures *outside* the control problem. This procedure falls short because control problems have a natural and highly non-uniform measure of belief quality: task-relevant errors propagate through time and limit performance. Consequently, it is possible to develop more robust and resource-efficient controllers by optimizing inference and expected utility together.

It’s worth noting that, while today’s machines struggle to design control strategies that account for the cost of thinking to reduce uncertainty, living organisms have already resolved this issue through millions of years of evolution [7, 8], so biological control systems may provide some guidance about intelligent control. Behavioral experiments consistently show the brain engages in probabilistic inference to transform sensory input into valuable conclusions that guide behavior [9, 10, 11]. Although the specifics of how neural circuits implement this cognitive process are debated, it is widely accepted that this is a resource-intensive process. To represent and process information, the brain uses action potentials (spikes) that consume a significant fraction of its total energy [12]. This representational constraint diminishes the animal’s ability to draw accurate conclusions and forces it to accept trade-offs: the brain can obtain more utility overall by sacrificing some task performance if doing so saves enough spikes. It is remarkable that despite operating within tight energy or computation budgets [13, 14], brains demonstrate exceptional control abilities in uncertain environments. Picture, for example, the spectacular precision of a peregrine falcon as it finds prey and estimates the trajectory of its deadly, high-speed dive, or the graceful agility of a hummingbird avoiding collisions as it traverses dense foliage in search of nectar-filled flowers.

This research aims to contribute to understanding fundamental principles that enable brains to balance solving complex control tasks against computational cost [15, 16]. In particular, we investigate how brain-like constrained agents solve sequential control problems based on partial observations and approximate inference. To do so, we develop a novel control framework that enables a meta-level rational agent to jointly optimize task performance (i.e., expected utility) along with the neural cost of representing beliefs (here, the cost of confidence) in a task involving continuous state and action spaces. By solving and analyzing this joint inference and control problem, we show how agents can obtain a family of strategies that reduce the costs of inference. Solutions in this family differ in their faulty assumptions about the world, each accepting some approximation errors while only incurring bounded costs on task performance.

## 2 Related work

Previous efforts to study how resource-constrained agents (brains and machines) reason under uncertainty can be classified as focusing on coding, inference, and control.

### Efficient coding.

A long history of research successfully explains how the brain represents sensory stimuli within constraints of limited coding resources. Many of these addressed sensory coding [17, 18, 19, 20, 21, 22]. A few considered uncertainty explicitly [23, 24], and one addressed efficient coding in the service of control [25]. Their scope is restricted to feedforward settings or overlook the impact of resource constraints in the inference process.

### Efficient inference for decision-making.

Studies such [26], [27], and [28] identify tasks where simple heuristics outperform complex reasoning mechanisms. Though these works succeed in finding settings where synthesizing more information does not necessarily yield superior performance, their scope is limited to problems involving a single, categorical decision. Studying the effects of efficient inference on control tasks that also require efficient coding is challenging, most works rely only on behavioral experiments for insights and do not delve into the computational implementation [29].

### Computationally Constrained rationality.

While the essence of control theory is balancing the trade-off between the costs and rewards of actions given constraints (e.g. time, dynamics, controls and sensing), few studies have explored how performance trades off against costs of internal computations that drive the controls. In general, constraints have two effects: they restrict the feasible subspace for control, and they induce additional uncertainty. This leads to two corresponding types of compensatory modifications that: change the policy/controller to preserve feasibility (*bounded rational control*) or change the inference/estimator to reduce uncertainty (*adaptive control*)[30].

Computationally constrained control has been previously studied using bounded rationality, which formalizes resource limitations as hard constraints the agent satisfies by restricting its policy to a feasible space, for instance, compensating risk incurred through sensing constraints using upper bounds on estimation error [31]. Our approach formulates a meta-level rational agent that jointly optimize utility and computational costs over both control and inference. Related work has been more limited in scope, for example one-step control problems [32], or restricting to internal actions that only modify inference [33], including truncating computation [34, 16, 30] and active sensing [35, 36]). The general meta-level constrained rationality in continuous spaces has remained an open problem addressed only conceptually [37, 38, 15]. Levine discusses how the full problem could be formulated in principle as structured variational inference [39], highlighting the need to structure and constrain the approximation to both parameterize costs and ensure the agent cannot control its inference in ways that decouple it from reality.

## 3 Methods

We mathematize how the brain transforms sensory evidence into useful actions as a Partially Observable Markov Decision Process (POMDP). In a POMDP, the agent interacts with an environment (world) over time, perceiving the world state through noisy observations and taking actions that change this state. The agent is rewarded based on the actions taken and the resulting state transitions, and its goal is to find the sequence of actions that maximizes total expected utility. Since the world state is not directly knowable, solving a POMDP requires keeping track of the relevant information distilled from the history of observations seen and actions executed so far. An efficient way to keep track of this information is to use recursive Bayesian inference to compress it into an evolving posterior probability distribution (belief) over the hidden world state. This belief helps the agent make decisions that maximize expected utility.

### 3.1 The neural cost of confidence

Copious behavioral evidence demonstrate that brains often reason probabilistically [9]; however, how neural activity represents probability distributions and implements operations of probabilistic inference remains debated. Two prominent hypotheses are the probabilistic population code (PPC) [40] and the neural sampling code [41, 42]; the former suggests that neural activity encodes natural parameters of posterior distributions, whereas the latter proposes that neural responses are samples from the represented distribution. In both cases, the encoded distributions get sharper and less variable as the number of action potentials (spikes) increases [40], [43]. Given that generating spikes imposes a significant metabolic load on the brain [12], we define the neural cost of decreasing state uncertainty as the expected number of action potentials a spiking neural network uses to implement recursive Bayesian inference and represent posterior distributions. In Appendix A, we detail how we calculate the spike count for PPCs and prove that, when the estimates are minimally biased, the spike count is lower bounded by the mutual information between hidden states and estimates.

### 3.2 Reasoning when confidence is costly

To identify fundamental principles that allow the brain to solve hard control problems under tight energy budgets that diminish its capability to decrease state uncertainty, we developed a version of stochastic control that accounts for the expected number of spikes required to implement recursive Bayesian inference and represent posterior distributions. In our framework, the agent optimizes this neural cost of inference *along* with the task performance; as a result, the agent finds a joint inference and control strategy that balances the cost of computing beliefs, the task performance, and the moving effort needed to compensate for estimation errors.

To isolate the effects of imposing a penalty on the cost of the inference process, we focus our study on the Linear Quadratic Gaussian (LQG) setting, which is a POMDP where the dynamics are linear, the sources of noise are Gaussian, and the state costs and action costs are both quadratic. Without our added cost of confidence, this setting can be solved analytically [44]. To be concrete, we study the performance of an agent that aims to minimize the deviation of a controllable state from a target (for example, a hummingbird extracting nectar from a flower, or an autonomous drone landing). We model this task using a linear dynamical system (Equation 1). For clarity, the development is explained using a one-dimensional system. However, the results are applicable to N-dimensional systems, as demonstrated in the results section.

(1) $$x_{t}=ax_{t-1}+bu_{t-1}+w_{t-1},\;y_{t}=x_{t}+v_{t}$$

where $x_{t}$ is the agent’s position (world state), a is the coefficient that determines how unstable the world is, b is the input matrix, $u_{t}$ is the action that changes the state, and $w_{t}$ is additive white Gaussian noise with variance q; the agent perceives this world through noisy observations $y_{t}$ corrupted by additive white Gaussian noise $v_{t}$ with variance r. The agent builds estimates ${\overset{ˆ}{x}}_{t}$ of the hidden world state as described below, and its actions are linear functions of this estimate: $u_{t}=l\;{\overset{ˆ}{x}}_{t}$, where l is a control gain.

To obtain ${\overset{ˆ}{x}}_{t}$, the agent computes an evolving posterior over the world, which we identify as the belief $b_{t}$, using recursive Bayesian inference:

$$b_{t}=p\left(x_{t}|y_{0},\cdots ,y_{t},u_{0},\cdots ,u_{t}\right)\propto p\left(y_{t}|x_{t}\right)\int p\left(x_{t}|x_{t-1},u_{t-1}\right)b_{t-1}dx_{t-1}$$

For our stationary LQG system, this recursive filtering leads to a Gaussian posterior at time t, with minimal sufficient statistics given by a mean ${\overset{ˆ}{x}}_{t}$ and a variance $\sigma_{t}^{2}$. The variance is the solution of the Ricatti equation

$$\sigma_{t}^{2}=\frac{\left(a^{2}\sigma_{t-1}^{2}\;+\;q\right)r}{a^{2}\sigma_{t-1}^{2}\;+\;q\;+\;r},$$

is independent of observations, and over time, it asymptotically approaches an equilibrium value, ${\overset{‾}{\sigma }}^{2}=kr$, that depends only on the system parameters Ω={a,q,r}. At this equilibrium, the mean can be expressed as an exponential weighted sum of past observations:

(2) $${\overset{ˆ}{x}}_{t}=\frac{(a\;+\;l)r}{a^{2}\sigma_{t-1}^{2}\;+\;q\;+\;r}{\overset{ˆ}{x}}_{t-1}+\frac{a^{2}\sigma_{t-1}^{2}\;+\;q}{a^{2}\sigma_{t-1}^{2}\;+\;q\;+\;r}y_{t}=\left(a+l\right)\left(1-k\right)\;{\overset{ˆ}{x}}_{t-1}+k\;y_{t}\;\;=k\sum_{i=0}^{t}\;((a+l)(1-k){)}^{i}\;y_{t-i}$$

The belief that results from using the actual system parameters Ω to marginalize out all the possible ways the world state might have changed from time t−1 to time t is optimal, i.e., its corresponding ${\overset{ˆ}{x}}_{t}$ minimizes mean squared error $E[{\left({\overset{ˆ}{x}}_{t}-x_{t}\right)}^{2}|y_{0:t}]$. Nevertheless, a resource-constrained agent (such as the brain that operates within a tight energy budget or a small device with limited computational capabilities) may not be able to afford computing and representing optimal beliefs.

To meet resource constraints, our meta-level, rational agent engages in the inference process described above, but this time using the wrong world parameters $\tilde{\Omega }=\{\tilde{a},\tilde{q},\tilde{r}\}$ that, instead of faithfully representing reality, are tailored to balance inference quality against the overall benefit that inferences provide in solving the control task. This leads to a larger — and thus cheaper — posterior variance and a mean given by a different exponential weighted sum on the sensory observations, that is:

(3) $${\overset{ˆ}{x}}_{t}=\frac{(\tilde{a}\;+\;l)\tilde{r}}{{\tilde{a}}^{2}\sigma_{t-1}^{2}\;+\;\tilde{q}\;+\;\tilde{r}}{\overset{ˆ}{x}}_{t-1}+\frac{{\tilde{a}}^{2}\sigma_{t-1}^{2}\;+\;\tilde{q}}{{\tilde{a}}^{2}\sigma_{t-1}^{2}\;+\;\tilde{q}\;+\;\tilde{r}}y_{t}=\alpha \;{\overset{ˆ}{x}}_{t-1}+\beta \;y_{t}=\beta \sum_{i=0}^{t}\alpha^{i}\;y_{t-i}$$

with $\alpha =(\tilde{a}+l)(1-\beta )$. The exponential weighted sum on the sensory observations (Equation 3 is an exponential filter; here, the temporal discount factor α determines how much of the past is worth remembering and the scaling factor β defines the units of the estimate ${\overset{ˆ}{x}}_{t}$.

Without resource constraints, a Kalman filter can compute beliefs that maximally reduce state uncertainty; this enables the agent to use estimates as surrogates of the hidden world states and, without loss of optimality, solve the control problem as if it were fully observable [44]. However, when the cost of decreasing uncertainty matters and optimal beliefs are not affordable, the agent can apply stronger control gains to counterbalance estimation errors. Consequently, imposing a penalty on the cost of inference couples the dynamics of the filter and the controller and breaks down the so-called separation principle, a fundamental property of LQG settings that allows the efficient computation of optimal solutions.

To find the joint filtering and control strategy that balances the cost of being in the wrong place, the cost of moving, and the cost of thinking to decrease uncertainty, our agent has to solve the following optimization problem:

(4) $$min{\;E}_{\Omega }\left[\sum_{t=0}^{T}\;c_{x}\;x_{t}^{2}+c_{u}\;u_{t}^{2}+c_{n}\left(I\left(x_{t};\;y_{t}\right)+I\left(x_{t};\;{\overset{ˆ}{x}}_{t}\right)\right)\right]$$

In the loss function, the mutual information $I\left(x_{t};y_{t}\right)+I\left(x_{t};{\overset{ˆ}{x}}_{t}\right)$ is a lower bound of the neural cost of confidence (see details in Appendix A), and the coefficients $c_{x}$, $c_{u}$, and $c_{n}$ penalize the agent for being far from the goal state $\left(x_{goal}=0\right)$, taking large actions, and synthesizing too much information, respectively.

Here we make a critical assumption: the expectation E is taken with respect to the probability distribution of trajectories $\tau =\left\{x_{0:T},\;y_{0:T},\;{\overset{ˆ}{x}}_{0:T},\;u_{0:T}\right\}$ that obey the dynamics determined by the *true* world model Ω, not the subjective one $\tilde{\Omega }$ used to integrate evidence. Why do we introduce this discrepancy between the model used for inference and the one used for control? The reason is that our agent only pays an internal cost for its inferences, not its controls. The agent can thus deliberately choose to suboptimally compress its belief information while observing the real consequences of its actions; this last allows optimizing the control gain to compensate for lossy compression.

To solve problem 4, we create an augmented system that describes the interactions among states, observations, estimates, and actions. In LQG settings, the observations are linear functions of the states, and actions are linear functions of the estimates; hence, the augmented system is fully described by the evolution of $z_{t}={\left[x_{t},u_{t}\right]}^{⊤}$:

$$z_{t}=\left[\begin{array}{cc} a & b\\ a\Psi & \Gamma +b\Psi \end{array}\right]z_{t-1}+\left[\begin{array}{c} w_{t-1}\\ \Psi \left(w_{t-1}+v_{t}\right) \end{array}\right]=Mz_{t-1}+\eta_{t-1}$$

where Ψ=lβ is an scaled control gain and $\Gamma =l\;\alpha \;l^{-1}$ is an invariant transformation of the temporal discount factor α. If $z_{t}$ can be stabilized using control, it reaches equilibrium. In equilibrium, $z_{t}∼𝒩(0,\Sigma )$, where Σ is a joint covariance matrix that summarizes the statistical dependencies among states, observations, estimates, and actions. Given Σ, it is straightforward to calculate the mutual information between the world state $x_{t}$ and the inferred estimate ${\overset{ˆ}{x}}_{t}$. In equilibrium, $I\left(x_{t};\overset{ˆ}{x}\right)$ equals the logarithm of the estimate’s relative precision, i.e., $I\left(x_{t};\overset{ˆ}{x}\right)=\frac{1}{2}{log}_{2}\left(\sigma_{\overset{ˆ}{x}}^{2}/\sigma_{\overset{ˆ}{x}|x}^{2}\right)$, where $\sigma_{\overset{ˆ}{x}}^{2}$ and $\sigma_{\overset{ˆ}{x}|x}^{2}$ are the marginal variance of the estimate and the conditional variance of the estimate given the state, respectively. Analogously, at the sensory input, the mutual information in equilibrium between world states and observations is $I\left(x_{t};y_{t}\right)=\frac{1}{2}{log}_{2}\left(\sigma_{y}^{2}/\sigma_{y|x}^{2}\right)$. Appendix A.5 provides more details on this derivation.

Thus, in equilibrium, problem 4 becomes:

(5) $$\underset{\Gamma ,\Psi }{min}\;\left\{c_{x}\sigma_{x}^{2}+c_{u}\sigma_{u}^{2}+c_{n}\frac{1}{2}\left({log}_{2}\frac{\sigma_{y}^{2}}{\sigma_{y|x}^{2}}+{log}_{2}\frac{\sigma_{\overset{ˆ}{x}}^{2}}{\sigma_{\overset{ˆ}{x}|x}^{2}}\right)\right\}$$

We solve problem 5 numerically. The optimal policy $\pi^{*}=\{\Gamma^{*},\Psi^{*}\}$ minimizes total loss and guarantees controllability (i.e., a transition matrix M with stable eigenvalues) and valid probability distribution p(τ) (i.e., a positive definite covariance matrix $\Sigma^{*}$).

## 4 Results

Problem5 is a non-convex optimization problem whose analytical solution is an intricate function, even for simple cases. We solve it numerically and confirm the validity of the solutions using simulations. By finding joint filtering and control strategies, π={Γ,Ψ}, that minimize both task and inference cost, we identify fundamental principles that brain-like resource constrained agents employ to rationally approximate inference for efficient control. Our findings provide insights into the decision-making processes of such agents and can inform the development of more robust and resource-efficient artificial controllers.

### 4.1 One way to be perfect, many ways to be wrong

**Figure 1:**
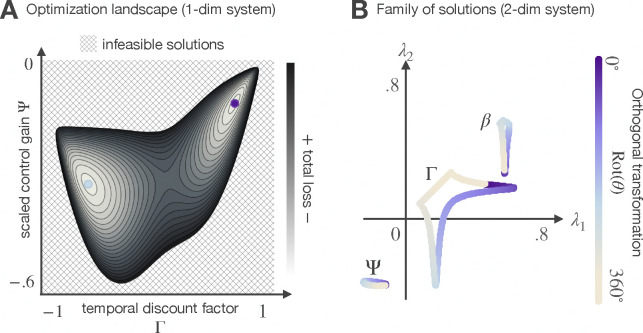
Landscape of the optimization problem. Without resource constraints, there is only one strategy {Γ,Ψ} that minimizes total loss. However, when the cost of confidence matters, the optimization landscape changes significantly. For resource-constrained agents, a family of resource-saving strategies can minimize total loss. These strategies are parameterized by a free orthogonal transformation (i.e., a rotation in the parameter space). For one-dimensional systems, {Γ,Ψ} are scalars that can only be moved along its single axis, either positively or negatively; hence, for one-dimensional systems, the optimization landscape has two global minima (panel ). In the multivariate context, {Γ,Ψ} are matrices and their transformations are specified by an axis of rotation and an angle of rotation. Panel **B** illustrates the infinite family of strategies that solve a two-dimensional task; the angle of rotation that relates family members is denoted using a color scheme. Each point in the figure corresponds to a combination in the family (note that we plot the eigenvalues of the matrices for visualization purposes). Conceptually, Γ represents an invariant transformation of the memory of the exponential filter (a parameter that determines how much of the past is worth remembering), β quantifies the extent to which the agent should focus on new information, and Ψ can be understood as a scaled control gain. As this figure shows, the members of the family trade off memory, attention to new evidence, and control effort in different ways to minimize total loss

Solving problem 5 implies finding the level of confidence that balances inference quality against the overall benefit the inference provides in solving the task. On the one hand, for an unconstrained agent, there is only one way to solve problem 5: use an accurate world model, perform optimal probabilistic inference to get precise estimates, and use them to drive controls as if they were fully observable states. In our framework, it is expensive to achieve this unique, maximally precise solution. On the other hand, there are infinitely many ways to be wrong. When computational and representational resources limit the agents capability to carry out optimal inference, the solution to problem 5 is a family of joint inference and control strategies parameterized by a free orthogonal transformation on the matrix parameters for inference and control (Appendix B). All members of this family produce identical costs, because they yield the same bounded-optimal covariance matrix $\Sigma^{*}$ whose entries fully characterize how the inference and control parameters affect the components of the loss function. Figure 1 illustrates the optimization landscape of an example 1-dimensional system (A) and the eigenvalues of the optimal parameters that solve a 2-dimensional example system (B).

**Figure 2:**
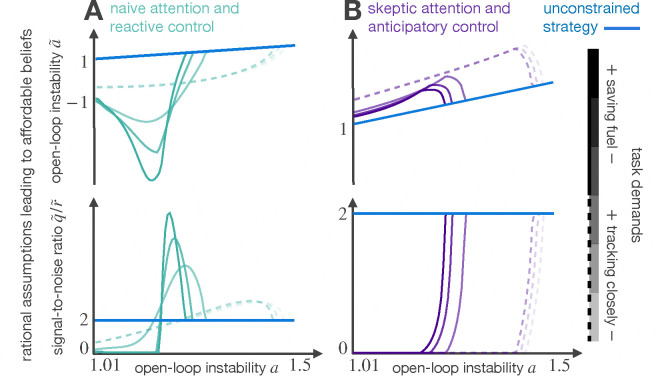
Different ways to interpret uncertainty. A) In easy tasks, modeling uncertainty as oscillations in a world with deterministic dynamics minimizes inference cost. In moderately difficult tasks, the noise is modeled as stronger oscillations in a stochastic world. Highly unstable worlds are fragile to model mismatch; the range of instability that can tolerate model mismatch changes according to task demands. B) An equivalent solution is to interpret the disturbances in the world as additional volatility.

The extreme behaviors of the family of solutions are: i) to be skeptical of incoming evidence and apply anticipatory control, and ii) to over rely on incoming evidence and apply reactive control. The first strategy interprets the disturbances in the world (that come from process and observation noise) as oscillations in the system response. The second strategy, in contrast, models the stochasticity as additional volatility. Figure 2 shows how these strategies adapt to changes in task demands.

### 4.2 Attention to incoming evidence

**Figure 3:**
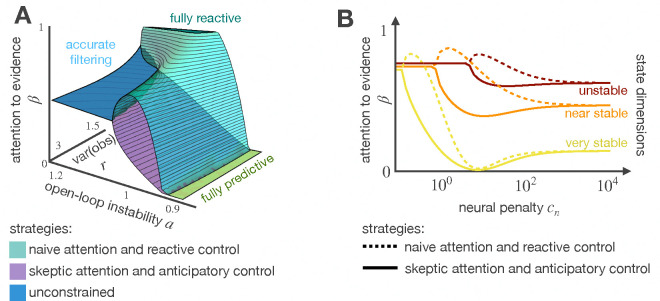
Evidence integration. Rational agents approximate inference based on world properties and the control objective. This leads to non-monotonical changes in the attention to new evidence (A). In multidimensional contexts, rational agents allocate their resources wisely by disregarding observations from stable directions and focusing on synthesizing optimal estimates in volatile directions where making mistakes would be devastating (B).

Unconstrained agents integrate incoming evidence by weighing observations and previous knowledge based on their statistical reliability. This strategy generates estimates that minimize mean squared error and is agnostic to the control objective. Rational agents, in contrast, determine how much of the past is worth to be remembered and how much attention new observations deserve based on the control objective and the penalty on the inference cost. As Figure 3 shows, the inference mechanism of rational agents goes through phase transitions: 1) Optimal in volatile worlds; in this regime, the estimates guarantee minimal mean squared error 2) Fully reactive when the agent is modeling uncertainty as oscillations, and the quality of the observations is good; here, the agent relies entirely on its observations to guide behavior. 3) Fully predictive in sufficiently stable worlds; in this case, the agent relies entirely on feedforward predictions and the damped behavior of the system dynamics to complete the task. 4) Custom-fit in worlds robust to model mismatch; here, the agent makes decisions based on suboptimal estimates that meet resource constraints. Figure 3. A further illustrates that an agent modeling uncertainty as oscillations pays more attention to incoming evidence compared to an agent modeling noise as additional volatility. Although both mechanisms of attention, i.e., being naive or skeptical of incoming observations, minimize total loss (problem57), the movement trajectories they induce are quite different; we discuss the differences in subsection 4.3.

To investigate how rational agents integrate evidence in multidimensional state spaces, we challenged an agent to stabilize a controllable state in a 3D space. The directions in this space varied in their stability: one is volatile, another is near-to-stable, and the third is completely stable. The signal-to-noise ratio, q/r, is the same regardless of the dimension. As shown in Figure 3B, rational agents allocate their resources wisely: even though the quality of the observations is the same in all three directions, rational agents choose to disregard the observations coming from the stable direction and focus on synthesizing optimal estimates in the volatile direction, where making mistakes can have devastating consequences.

### 4.3 Moving more to think less

**Figure 4:**
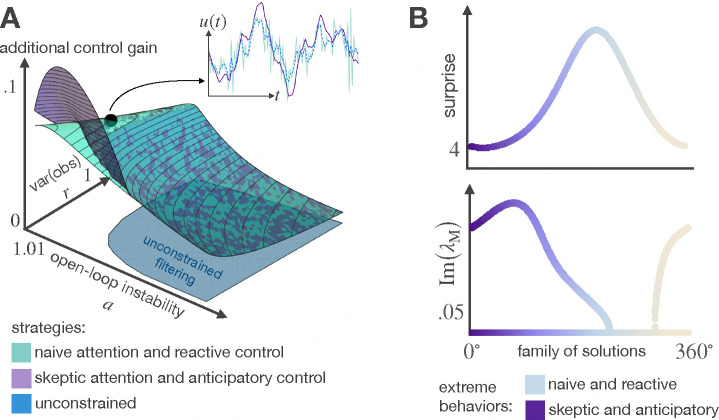
Moving more to think less. Rational agents apply stronger control gains compared to unconstrained agents. A higher control gain can either offset the errors resulting from suboptimal inference or make optimal beliefs affordable by reducing state variance (A). The differences in the movement trajectories of naive and skeptical agents can be explained by studying the level of surprise (the variance of the difference between observations $y_{t}$ and predicted observations $(\tilde{a}+l)\;{\overset{ˆ}{x}}_{t}$) and the eigenvalues of the transition matrix M that describes how states and actions jointly evolve. Naive agents experience the most surprise and require reactive control to compensate for estimation errors. In contrast, the anticipatory control of skeptical agents induces oscillations in the system response (complex eigenvalues $\lambda_{M}$) that leads to smoother trajectories.

Compared to unconstrained agents, rational agents always apply stronger control gains. As shown in Figure 4 in regions where the rational choice is to filter suboptimally, the agent uses a higher control gain to compensate for the errors that come with suboptimal estimates; the magnitude is even higher when the agents behave fully reactive to incoming evidence. Interestingly, a stronger control gain is also applied when the filtering mechanism performs optimally; in this case, the agent uses control to make beliefs relatively cheaper by decreasing state variance. It is worth noting that the trajectories generated by naive and skeptical agents have distinct differences. On the one hand, the skeptical agent prioritizes the predictions of its world model over new observations and assumes deterministic dynamics, attributing any uncertainty in the world to additional instability; this assumption leads to actual oscillations in the system response, resulting in smooth and natural-looking movement trajectories. On the other hand, the naive agent, who gives more importance to new observations than predictions, requires frenetic movements to compensate for the suboptimality of its beliefs.

### 4.4 Generalization to nonlinear settings - Sigway

We applied our framework to solve the problem of stabilizing an inverted pendulum on a cart. The system’s schematic is shown in panel A. The system is inherently unstable and is described by nonlinear dynamics. To achieve stabilization, the agent must determine the sequence of actions (accelerate/decelerate) that guide the state s=[cartpositionx,velocityν,pendulumangleθ,angularvelocityω] to the goal state $s^{*}=[0,\;0,\;0,\;0]$.

Due to the addition of Gaussian process and observation noise, the system’s state is hidden. Therefore, the agent must synthesize beliefs to guide actions that minimize total expected average cost. For this experiment, we linearized the system’s dynamics and assume the following parameter values: pendulum mass m=1, cart mass W=5, pendulum length l=2, gravitational acceleration g=10, Gaussian process and observation noise with covariances Q=I and R=diag(0.05,0.3,0.05,.3), state penalty $c_{x}=diag\left(10^{-4},10^{-4},\;10,\;10\right)$, action penalty $c_{u}=.1I$, and inference penalty $c_{n}=500$.

Panel **B** displays the trajectories of states and actions when the agent solves the problem using classic LQG (dashed blue), and bounded rational control (purple and cyan). The behavior in red represents the system’s performance before any strategy is implemented. These trajectories, along with the expected average costs achieved by the competing strategies (reported in Table 1), indicate that the insights gained from studying how to control generic random walks under resource constraints still hold true for more complex systems. As predicted, two strategies minimize total loss when the actions are scalars. One strategy relies heavily on incoming evidence and requires rapid movements to account for estimation errors (cyan). The other strategy uses the world model to anticipate estimation errors and makes corrections using smoother movements (purple). The actions’ trajectories confirm another prediction: rational agents move more to reduce state variance, allowing them to collect new observations that are relatively cheaper to compress into a belief. This reduction in state variance is particularly noticeable along the dimension that encodes the cart position.

## 5 Discussion

We present a novel approach to stochastic control where the resources invested to decrease uncertainty are optimized along with the task performance. In our framework, the estimation error is a metacontrol variable the agent can modulate. This couples the filtering and control dynamics and creates a trade-off: an agent can obtain more utility overall by sacrificing some task performance, if doing so saves enough bits in the inference process. In the multivariate context, we found that this trade-off expands the principle of minimal intervention in control, which states that control should be exerted only on deviations that worsen the objective. Here, instead of just minimizing action costs, our agents also pay inference costs, choosing what to pay based in part on task demand, attenuating or discarding those dimensions with relatively small consequences.

Our findings suggest that brain-like, resource-constrained agents model less of the world on purpose to lessen the burden of optimal inference. Depending on the task demands, the rational strategy that solves the joint inference and control problem goes through phase transitions, switching from a costly but optimal inference to a resource-saving solution that misestimates the world’s stability. We found that there is a family of resource-saving solutions that perform equally well in balancing task performance, inference quality, and moving effort but differ in the way the agent models the uncertainty in the world, pays attention to incoming observations, counteracts estimation errors, and minimizes the surprises caused by using a mismatched world model to integrate evidence. The brain may use this treat to select the family member that best generalizes across tasks and meets additional constraints imposed by the environment or physical capabilities.

Even though our agents pay a cost for confidence, they do not explicitly reap its full benefits in our current demonstrations. This is because the LQG control setting is peculiar in being solvable non-probabilistically by altering how we weigh and use incoming evidence, without appealing to subjective confidence at all. However, the real benefit of the probabilistic framework enters when the system changes over time. An agent facing such a task has two choices. First, without some neural representation of the changing system state, the non-probabilistic optimization would need to solve the control problem for each context separately, anew. Second, it could directly represent the changing context state, and use this to optimally modulate how it weighs evidence and adjust its controls while accounting for the cost of this representation. In a slowly adapting linear system, this second option is computationally equivalent to our probabilistic framing, but without the benefits of interpretability. Our approach thus offers a principled way of specifying an agent that generalizes gracefully, while minimizing representational costs. In future work, we will directly evaluate the benefits of maintaining a representation of confidence in an environment with dynamic signal-to-noise ratio. This will be closer to real-world task demands for which brains evolved, so it will enable us to apply our conceptual framework to make specific predictions for practical experiments.

### Broader impacts.

Understanding the neural foundations of thought would have major impacts on human life, through neurology, brain-computer interfaces, artificial intelligence, and social communication. Our work aims to refine the theoretical foundations for such understanding. Thoughtless or naive application of these scientific advances could lead to unanticipated consequences and increase social inequality.

## Figures and Tables

**Figure 5:**
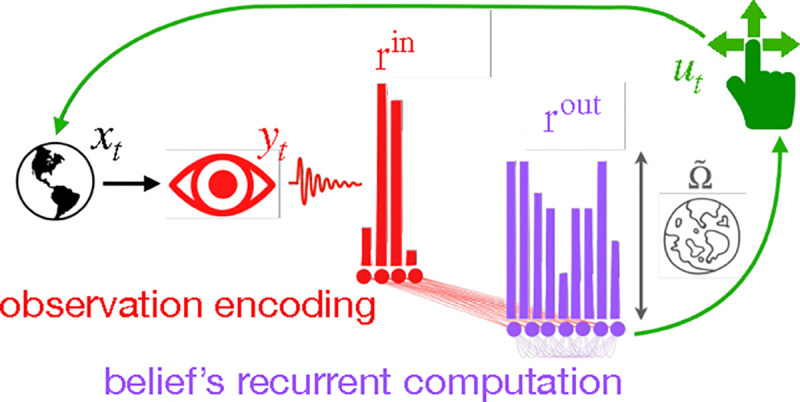
Spiking Neural Network. Recursive Bayesian inference is implemented neurally using a dynamic Probabilistic Population Code: linear projections of spiking activity approximate the natural parameters of the likelihood and the belief.

## A The neural cost of decreasing uncertainty

In this Appendix, we detail how we quantify the number of action potentials a spiking neural network uses to perform recursive Bayesian inference. Then, we prove that, when the estimates are minimally biased, the spike count is lower bounded by the mutual information between hidden states and estimates.

## A.1 The spikes a neural circuit needs to perform recursive Bayesian inference

To determine how many spikes a neural circuit requires to encode observations and beliefs, we implement a spiking neural network based on the model described in [45]; we then calculate the expected number of spikes this network requires to carry out recursive Bayesian inference.

As in [45], we assume that the brain represents probabilities over the world state $x_{t}$ using a Probabilistic Population Code (PPC), which is a distributed neural representation that support evidence integration through linear operations and marginalization through nonlinear operations. Accordingly, as illustrated in Figure 5, spike trains, $r^{in}$, emitted by an input layer of neurons with Poisson-like response variability encode observations $y_{t}$. Next, neural activity $r^{in}$ feeds a recurrent layer whose firing activity, $r^{\text{out}}∼Poi\left(\nu_{t}\right)$, encodes the belief $b_{t}=𝒩\left({\overset{ˆ}{x}}_{t},{\overset{‾}{\sigma }}^{2}\right)=𝒩\left(\alpha {\overset{ˆ}{x}}_{t-1}+\beta y_{t},\tilde{r}\beta \right)$ that the agent would obtain by implementing recursive Bayesian inference based on its model of the world $\tilde{\Omega }=\{\tilde{a},\tilde{q},\tilde{r}\}$, last observation seen, and last action executed. Finally, the agent decodes the world state using linear projections of the activity $\nu_{t}$, that is:

(6)
$$\xi \cdot \nu_{t}=\frac{\alpha {\overset{ˆ}{x}}_{t-1}\;+\;\beta y_{t}}{\tilde{r}\;\beta }=\frac{{\overset{ˆ}{x}}_{t}}{{\overset{‾}{\sigma }}^{2}}$$

(7)
$$\zeta \cdot \nu_{t}=\frac{1}{\tilde{r}\;\beta }=\frac{1}{{\overset{‾}{\sigma }}^{2}}$$

An expression for the firing rate $\nu_{t}$ that is consistent with the decoding mechanism (Equations 6 and 7) is:

(8)
$$\nu_{t}^{\text{out}}=\frac{\zeta }{\left\|\zeta \right\|}\frac{1}{\tilde{r}\;\beta }+\frac{\xi }{\left\|\xi \right\|}(\alpha (\xi \cdot r_{t-1}^{\text{out}})+\xi \cdot r_{t}^{in})+c\cdot 1$$

Equation 8 holds for arbitrary vectors ξ and ζ as long as they are orthogonal to each other and to the vector 1. We use $\xi_{k}=cos\left(\frac{2\pi k}{N}\right)/N$ and $\zeta_{k}=cos\left(\frac{4\pi k}{N}\right)/N$, where N is the number of neurons in the recurrent layer. The term c⋅1 in Equation 8 is an offset that ensures positive firing rates.

Given that $r^{\text{out}}∼Poi\left(\nu_{t}\right)$, the expected spike count in the recurrent layer is $E\left[1\cdot r_{t}^{\text{out}}\right]=1\cdot \nu_{t}=Nc$. Thus, the minimum number of spikes the neural circuit requires to encode observations and synthesize beliefs is proportional to the minimum value $c_{\text{min}}$ that ensures positive firing rates. By calculating the derivative of $\nu_{t}$ with respect to index k and identifying the critical points of the function, we found the expected value of $c_{min}$:

(9)
$$E\left[c_{min}\right]=\frac{8\;+\;\sigma_{\overset{ˆ}{x}}^{2}}{\tilde{r}\;\beta }$$

where $\sigma_{\overset{ˆ}{x}}^{2}$ is the marginal variance of the estimate. Simulations confirm this result.

## A.2 The bias-variance trade-off in recursive Bayesian inference

We calculate the bias of the exponential filter using the bias-variance decomposition of the error $e_{t}={\overset{ˆ}{x}}_{t}-x_{t}$. The mean squared error (MSE) given the history of observations up to time t is:

(10)
$$E[{\left({\overset{ˆ}{x}}_{t}-x_{t}\right)}^{2}|y_{0:t}]=var\left({\overset{ˆ}{x}}_{t}-x_{t}|y_{0:t}\right)+E{\left[{\overset{ˆ}{x}}_{t}-x_{t}|y_{0:t}\right]}^{2}\;\;=var\left(x_{t}|y_{0:t}\right)+var\left({\overset{ˆ}{x}}_{t}|y_{0:t}\right)-2\;cov\left(x_{t},{\overset{ˆ}{x}}_{t}|y_{0:t}\right)+E{\left[{\overset{ˆ}{x}}_{t}-x_{t}|y_{0:t}\right]}^{2}\;\;=var\left(x_{t}|y_{0:t}\right)+0-2(E\left[x_{t},{\overset{ˆ}{x}}_{t}|y_{0:t}\right]-E\left[x_{t}|y_{0:t}\right]E\left[{\overset{ˆ}{x}}_{t}|y_{0:t}\right])+E{\left[{\overset{ˆ}{x}}_{t}-x_{t}|y_{0:t}\right]}^{2}\;\;=var\left(x_{t}|y_{0:t}\right)+E{\left[{\overset{ˆ}{x}}_{t}-x_{t}|y_{0:t}\right]}^{2}\;\;=var\left(x_{t}|y_{0:t}\right)+E{\left[\beta \sum_{i=0}^{t}\;\alpha^{i}y_{t-i}-x_{t}|y_{0:t}\right]}^{2}\;\;=var\left(x_{t}|y_{0:t}\right)+E{\left[\beta \sum_{i=0}^{t}\;((\tilde{a}+l)(1-\beta ){)}^{i}y_{t-i}-x_{t}|y_{0:t}\right]}^{2}$$

Equation 10 shows that errors in the estimates of the exponential filter stem from two sources: the true posterior variance $var\left(x_{t}|y_{0:t}\right)$ and the bias, i.e., discrepancies arising from using a world model $\tilde{\Omega }$ that diverges from reality.

## A.3 Minimally biased estimates

Problem 5 hides a scaling degeneracy. In LQG settings, the actions are linear functions of the estimates, that is

$$u_{t}=l\;{\overset{ˆ}{x}}_{t}=l\;\beta \sum_{i=0}^{t}\alpha^{i}\;y_{t-i}$$

Due to this property, the effects of the scaling factor β can be reverted by the control gain l, and vice-versa. To mitigate this degeneracy, we constrain β to serve as the scaling factor that yields minimally biased estimates, i.e.,

(11)
$$\beta =arg\;\underset{s}{min}\;E{\left[s\sum_{i=0}^{t}\;((\tilde{a}+l)(1-s){)}^{i}y_{t-i}-x_{t}|y_{0:t}\right]}^{2}$$

when the world model matches reality, the solution to problem 11 is the classic Kalman gain and the bias is 0.

## A.4 Recovering world models from joint filtering and control strategies

In our framework, the agent builds world models $\tilde{\Omega }=\{\tilde{a},\tilde{q},\tilde{r}\}$ that are tailored to balance inference quality against the overall benefit inferences provide in solving the control task. The belief that results from using $\tilde{\Omega }$ to marginalize out all the possible ways the world state might have changed from time t−1 to time t is $b_{t}=𝒩(\beta \sum_{i=0}^{t}\;\alpha^{i}y_{t-i},\tilde{r}\;\beta )$. If $\tilde{\Omega }$ faithfully represented reality,

(12)
$$\alpha =(\tilde{a}+l)(1-\beta )$$

(13)
$$\beta =\frac{{\tilde{a}}^{2}\;-\;\tilde{q}/r\;-\;1\;+\;\sqrt{{\tilde{a}}^{4}\;+\;2{\tilde{a}}^{2}\;(\tilde{q}/\tilde{r}\;-\;1)\;+\;(\tilde{q}/\tilde{r}\;+\;1{)}^{2}}}{2\;{\tilde{a}}^{2}}$$

(14)
$$\tilde{r}\;\beta =MSE$$

Once problem 5 is solved, Equations 12, 13, and 14 allow to extract the agent’s world model.

## A.5 The mutual information between states and inferences is a lower bound of the spike count

The mutual information between the world state $x_{t}$ and the estimate ${\overset{ˆ}{x}}_{t}$ is:

(15)
$$I(x;\overset{ˆ}{x})=S_{x}+S_{\overset{ˆ}{x}}-S_{x\overset{ˆ}{x}}$$

where $S_{x}$ is the entropy of the state, $S_{\overset{ˆ}{x}}$ is the entropy of the estimate, and $S_{x\overset{ˆ}{x}}$ is the entropy of the joint probability distribution of states and estimates. Thus:

(16)
$$I(x;\overset{ˆ}{x})=\frac{1}{2}log\frac{\left|\sigma_{x}^{2}||\sigma_{\overset{ˆ}{x}}^{2}\right|}{\left|\sigma_{x}^{2}||\sigma_{\overset{ˆ}{x}}^{2}\;-\;\sigma_{x\overset{ˆ}{x}}^{2}\sigma_{x}^{-2}\sigma_{\overset{ˆ}{x}x}^{2}\right|}=\frac{1}{2}log\frac{\sigma_{\overset{ˆ}{x}}^{2}}{\sigma_{\overset{ˆ}{x}|x}^{2}}$$

with |⋅| denoting the determinant. When the scaling factor β sets the estimate in the correct units of the world (i.e., the estimates are minimally biased), Equation 16 becomes:

(17)
$$I(x;\overset{ˆ}{x})=\frac{1}{2}log\frac{\sigma_{x}^{2}}{\sigma_{e}^{2}}$$

where $\sigma_{e}^{2}$ is the marginal variance of the error $e_{t}={\overset{ˆ}{x}}_{t}-x_{t}$.

Equation 17 is a lower bound of the spike count (Equation 9) because:

(18)
$$E\left[c_{min}\right]=\frac{8\;+\;\sigma_{\overset{ˆ}{x}}^{2}}{\tilde{r}\;\beta }\;=\frac{8\;+\;\sigma_{\overset{ˆ}{x}}^{2}}{MSE}$$

(19)
$$\;=\frac{8\;+\;\sigma_{\overset{ˆ}{x}}^{2}}{\sigma_{e}^{2}}\;=\frac{8}{\sigma_{e}^{2}}+\frac{\sigma_{x}^{2}\;+\;\sigma_{e}^{2}}{\sigma_{e}^{2}}$$

(20)
$$\begin{array}{l} >\frac{\sigma_{x}^{2}}{\sigma_{e}^{2}}\\ >\frac{1}{2}log\frac{\sigma_{x}^{2}}{\sigma_{e}^{2}} \end{array}$$

In this reasoning, equality 18 follows directly from Equation 14 and equality 19 holds because, in equilibrium, the mean of the estimation error is 0.

## B Family of equivalent solutions for computationally constrained control

To find the family of equivalent solutions to our computationally constrained optimization problem, we study the covariance over the joint sequence of observations and actions that minimizes the loss function, $\Sigma^{*}$. Given that $u_{t}=\Gamma \;u_{t-1}+\Psi \;y_{t},\Sigma^{*}$ can be written as:

(21)
$$\left[\begin{array}{cc} \Sigma_{y} & \Sigma_{yu}^{⊤}\\ \Sigma_{yu} & \Sigma_{u} \end{array}\right]=\left[\begin{array}{cc} \Sigma_{y} & {\left(\Gamma \;\Sigma_{yu_{-}}+\Psi \;\Sigma_{y}\right)}^{⊤}\\ \Gamma \;\Sigma_{yu_{-}}+\Psi \;\Sigma_{y} & \Gamma \;\Sigma_{u_{-}}\;\Gamma^{⊤}+\Gamma \;\Sigma_{yu_{-}}\;\Psi^{⊤}+\Psi \;\Sigma_{yu_{-}}^{⊤}{\;\Gamma }^{⊤}+\Psi \;\Sigma_{y}\;\Psi^{⊤} \end{array}\right]$$

where $u_{-}=u_{t-1}$. By solving equation 21 element-wise, we find the family of strategies π={Γ,Ψ} that yield the same optimal covariance $\Sigma^{*}$. From the off-diagonal blocks we get:

(22)
Ψ=F−Γℱ

where $F=\Sigma_{yu}\Sigma_{y}^{-1}$ is the best linear predictor of u given y, and $ℱ=\Sigma_{yu_{-}}\Sigma_{y}^{-1}$ is the best linear predictor of $u_{-}$ given y. Using 22, we derive Γ from the diagonal blocks as follows:

(23)
$$\Sigma_{u}=\Gamma \left(\Sigma_{u_{-}}-ℱ\;\Sigma_{yu_{-}}-\Sigma_{yu_{-}}^{⊤}{ℱ}^{⊤}+ℱ\;\Sigma_{y}{ℱ}^{⊤}\right)\;\Gamma^{⊤}\;\;+\;\Gamma \;\left(\Sigma_{yu_{-}}^{⊤}F^{⊤}-ℱ\;\Sigma_{y}F^{⊤}\right)+{\left(\Sigma_{yu_{-}}^{⊤}F^{⊤}-ℱ\;\Sigma_{y}F^{⊤}\right)}^{⊤}\Gamma^{⊤}+F\;\Sigma_{y}F^{⊤}\;\;=\Gamma \;\Sigma_{\Delta_{u_{-}}}\Gamma^{⊤}-\Gamma \;\Sigma_{y\Delta_{u_{-}}}F^{⊤}-F\;\Sigma_{y\Delta_{u_{-}}}^{⊤}\Gamma^{⊤}+\Sigma_{\overset{ˆ}{u}}\;\;=(\Gamma -\Sigma_{y\Delta_{u_{-}}}^{⊤}\Sigma_{\Delta_{u_{-}}}^{-1})\Sigma_{\Delta_{u_{-}}}{\left(\Gamma -\Sigma_{y\Delta_{u_{-}}}^{⊤}\Sigma_{\Delta_{u_{-}}}^{-1}\right)}^{⊤}-\Sigma_{y\Delta_{u_{-}}}^{⊤}\Sigma_{\Delta_{u_{-}}}^{⊤}\Sigma_{y\Delta_{u_{-}}}^{⊤}+\Sigma_{\overset{ˆ}{u}}$$

where $\Sigma_{\Delta_{u_{-}}}$ is the covariance of the difference between the previous action and its predicted value given $y,\Sigma_{y\Delta_{u_{-}}}$ is the covariance between the above mentioned difference and the observation, and $\Sigma_{\overset{ˆ}{u}}$ is the covariance of the predicted, current action given y. Equation 23 takes the form of a similarity transform between $\left(\Gamma -\Sigma_{y\Delta_{u_{-}}}^{⊤}\Sigma_{\Delta_{u_{-}}}^{-1}\right)$ and $\Sigma_{\Delta_{u_{-}}}$ that squeezes the control-state representation. Note that the similarity relation allows a free orthogonal transform on the joint space, which produces a family of possible solutions:

$$\Sigma_{u}-\Sigma_{\overset{ˆ}{u}}+\Sigma_{y\Delta_{u_{-}}}^{⊤}\Sigma_{\Delta_{u_{-}}}^{⊤}\Sigma_{y\Delta_{u_{-}}}^{⊤}=D\sqrt{\Lambda }\Theta \Theta^{⊤}{\sqrt{\Lambda }}^{⊤}D^{⊤}\;\;=(\Gamma -\Sigma_{y\Delta_{u_{-}}}^{⊤}\Sigma_{\Delta_{u_{-}}}^{-1})\Sigma_{\Delta_{u_{-}}}^{\frac{1}{2}}\Sigma_{\Delta_{u_{-}}}^{\frac{T}{2}}{\left(\Gamma -\Sigma_{y\Delta_{u_{-}}}^{⊤}\Sigma_{\Delta_{u_{-}}}^{-1}\right)}^{⊤}$$

Therefore

(24)
$$\Gamma =D\sqrt{\Lambda }\Theta \Sigma_{\Delta_{u_{-}}}^{-\frac{1}{2}}+\Sigma_{y\Delta_{u_{-}}}^{⊤}\Sigma_{\Delta_{u_{-}}}^{-1}$$

Here, $D\Lambda D^{⊤}$ is the eigendecomposition of $\Sigma_{u}-\Sigma_{\overset{ˆ}{u}}+\Sigma_{y\Delta_{u_{-}}}^{⊤}\Sigma_{\Delta_{u_{-}}}^{-⊤}\Sigma_{y\Delta_{u_{-}}}^{⊤}$ and Θ is a rotation matrix that parameterizes a family of solutions with equal covariance, and therefore equal performance on the task for which they are optimized. These solutions differ, however, in their temporal structure, and thus they will generalize differently to novel tasks.

## References

[1] Kochenderfer Mykel J, Wheeler Tim A, and Wray Kyle H. Algorithms for decision making. MIT press, 2022.

[2] Ghavamzadeh Mohammad, Mannor Shie, Pineau Joelle, Tamar Aviv, Bayesian reinforcement learning: A survey. Foundations and Trends^®^ in Machine Learning, 8(5–6):359–483, 2015.

[3] Kurniawati Hanna. Partially observable markov decision processes and robotics. Annual Review of Control, Robotics, and Autonomous Systems, 5:253–277, 2022.

[4] Shuvaev Sergey A, Tran Ngoc B, Stephenson-Jones Marcus, Li Bo, and Koulakov Alexei A. Neural networks with motivation. Frontiers in Systems Neuroscience, 14:609316, 2021. 33536879 10.3389/fnsys.2020.609316 PMC7848953

[5] Barber David. Bayesian reasoning and machine learning. Cambridge University Press, 2012.

[6] Papadimitriou Christos H and Tsitsiklis John N. The complexity of markov decision processes. Mathematics of operations research, 12(3):441–450, 1987.

[7] Madhav Manu S and Cowan Noah J. The synergy between neuroscience and control theory: the nervous system as inspiration for hard control challenges. Annual Review of Control, Robotics, and Autonomous Systems, 3:243–267, 2020.

[8] Padamsey Zahid, Katsanevaki Danai, Dupuy Nathalie, and Nathalie L Rochefort. Neocortex saves energy by reducing coding precision during food scarcity. Neuron, 110(2):280–296, 2022. 34741806 10.1016/j.neuron.2021.10.024 PMC8788933

[9] Knill David C and Richards Whitman. Perception as Bayesian inference. Cambridge University Press, 1996.

[10] Knill David C and Pouget Alexandre. The bayesian brain: the role of uncertainty in neural coding and computation. TRENDS in Neurosciences, 27(12):712–719, 2004. 15541511 10.1016/j.tins.2004.10.007

[11] Sanders Honi, Wilson Matthew A, and Gershman Samuel J. Hippocampal remapping as hidden state inference. Elife, 9:e51140, 2020. 32515352 10.7554/eLife.51140 PMC7282808

[12] Attwell David and Laughlin Simon B. An energy budget for signaling in the grey matter of the brain. Journal of Cerebral Blood Flow & Metabolism, 21(10):1133–1145, 2001. 11598490 10.1097/00004647-200110000-00001

[13] Padamsey Zahid and Rochefort Nathalie L. Paying the brain’s energy bill. Current Opinion in Neurobiology, 78:102668, 2023. 36571958 10.1016/j.conb.2022.102668

[14] Vul Edward, Goodman Noah, Griffiths Thomas L, and Tenenbaum Joshua B. One and done? optimal decisions from very few samples. Cognitive science, 38(4):599–637, 2014. 24467492 10.1111/cogs.12101

[15] Gershman Samuel J, Horvitz Eric J, and Tenenbaum Joshua B. Computational rationality: A converging paradigm for intelligence in brains, minds, and machines. Science, 349(6245):273–278, 2015. 26185246 10.1126/science.aac6076

[16] Kool Wouter, Gershman Samuel J, and Cushman Fiery A. Planning complexity registers as a cost in metacontrol. Journal of cognitive neuroscience, 30(10):1391–1404, 2018. 29668390 10.1162/jocn_a_01263

[17] Attneave Fred. Some informational aspects of visual perception. Psychological review, 61(3):183, 1954. 13167245 10.1037/h0054663

[18] Barlow Horace B Possible principles underlying the transformation of sensory messages. Sensory communication, 1(01):217–233, 1961.

[19] Laughlin Simon. A simple coding procedure enhances a neuron’s information capacity. Zeitschrift für Naturforschung c, 36(9–10):910–912, 1981. 7303823

[20] Van Hateren J Hans. Spatiotemporal contrast sensitivity of early vision. Vision research, 33(2):257–267, 1993. 8447098 10.1016/0042-6989(93)90163-q

[21] Pitkow Xaq and Meister Markus. Decorrelation and efficient coding by retinal ganglion cells. Nature neuroscience, 15(4):628–635, 2012. 22406548 10.1038/nn.3064 PMC3725273

[22] Wei Xue-Xin and Stocker Alan A. A bayesian observer model constrained by efficient coding can explain’anti-bayesian’percepts. Nature neuroscience, 18(10):1509–1517, 2015. 26343249 10.1038/nn.4105

[23] Park Il Memming and Pillow Jonathan W. Bayesian efficient coding. BioRxiv, page 178418, 2017.

[24] Grujic Nikola, Brus Jeroen, Burdakov Denis, and Polania Rafael. Rational inattention in mice. Science advances, 8(9):eabj8935, 2022. 35245128 10.1126/sciadv.abj8935 PMC8896787

[25] Alex K Susemihl Ron Meir, and Opper Manfred. Optimal neural codes for control and estimation. Advances in neural information processing systems, 27, 2014.

[26] Gigerenzer Gerd and Brighton Henry. Homo heuristicus: Why biased minds make better inferences. Topics in cognitive science, 1(1):107–143, 2009. 25164802 10.1111/j.1756-8765.2008.01006.x

[27] Tavoni G, Doi T, Pizzica C, Balasubramanian V, and Gold JI. The complexity dividend: when sophisticated inference matters. biorxiv, 563346, 2019.

[28] Binz Marcel, Samuel J Gershman Eric Schulz, and Endres Dominik. Heuristics from bounded meta-learned inference. Psychological review, 2022. 10.1037/rev0000330 34990160

[29] Ho Mark K, Abel David, Correa Carlos G, Littman Michael L, Cohen Jonathan D, and Griffiths Thomas L. People construct simplified mental representations to plan. Nature, 606(7912):129–136, 2022. 35589843 10.1038/s41586-022-04743-9

[30] Bertsekas D.. A Course in Reinforcement Learning. Athena Scientific, 2023.

[31] Pacelli Vincent and Majumdar Anirudha. Robust control under uncertainty via bounded rationality and differential privacy. In 2022 International Conference on Robotics and Automation (ICRA), pages 3467–3474. IEEE, 2022.

[32] Gershman Samuel and Wilson Robert. The neural costs of optimal control. Advances in neural information processing systems, 23, 2010.

[33] Fox Roy and Tishby Naftali. Bounded planning in passive pomdps. arXiv preprint arXiv:1206.6405, 2012.

[34] Ortega Pedro A, Braun Daniel A, Dyer Justin, Kim Kee-Eung, and Tishby Naftali. Informationtheoretic bounded rationality. arXiv preprint arXiv:1512.06789, 2015.

[35] Młynarski Wiktor F and Hermundstad Ann M. Adaptive coding for dynamic sensory inference. eLife, 7:e32055, jul 2018. 29988020 10.7554/eLife.32055 PMC6039184

[36] Yang Scott Cheng-Hsin, Wolpert Daniel M, and Lengyel Máté. Theoretical perspectives on active sensing. Current Opinion in Behavioral Sciences, 11:100–108, 2016. Computational modeling. 10.1016/j.cobeha.2016.06.009 PMC6116896 30175197

[37] Horvitz Eric J. and Barry Matthew. Display of information for time-critical decision making, 2013.

[38] Braun Daniel A, Ortega Pedro A, Theodorou Evangelos, and Schaal Stefan. Path integral control and bounded rationality. In 2011 IEEE symposium on adaptive dynamic programming and reinforcement learning (ADPRL), pages 202–209. IEEE, 2011.

[39] Levine Sergey. Reinforcement learning and control as probabilistic inference: Tutorial and review, 2018.

[40] Ma Wei Ji, Beck Jeffrey M, Latham Peter E, and Pouget Alexandre. Bayesian inference with probabilistic population codes. Nature neuroscience, 9(11):1432–1438, 2006. 17057707 10.1038/nn1790

[41] Hoyer Patrik and Hyvärinen Aapo. Interpreting neural response variability as monte carlo sampling of the posterior. Advances in neural information processing systems, 15, 2002.

[42] Fiser József, Berkes Pietro, Orbán Gergõ, and Lengyel Máté. Statistically optimal perception and learning: from behavior to neural representations. Trends in cognitive sciences, 14(3):119–130, 2010. 20153683 10.1016/j.tics.2010.01.003 PMC2939867

[43] Kutschireiter Anna, Surace Simone Carlo, Sprekeler Henning, and Pfister Jean-Pascal. Nonlinear bayesian filtering and learning: a neuronal dynamics for perception. Scientific reports, 7(1):8722, 2017. 28821729 10.1038/s41598-017-06519-y PMC5562918

[44] Bertsekas Dimitri. Dynamic programming and optimal control: Volume I, volume 4. Athena scientific, 2012.

[45] Beck Jeffrey M, Latham Peter E, and Pouget Alexandre. Marginalization in neural circuits with divisive normalization. Journal of Neuroscience, 31(43):15310–15319, 2011. 22031877 10.1523/JNEUROSCI.1706-11.2011 PMC3230133

